# Prevalence and frequency of arrhythmias and electrocardiographic abnormalities in Pakistan: An outpatient ECG-based study

**DOI:** 10.1016/j.hroo.2024.12.017

**Published:** 2025-01-16

**Authors:** Aiysha Nasir, Armughan Tauheed Farooqi, Saadia Sattar, Yawer Saeed

**Affiliations:** Section of Cardiology, Department of Medicine, Aga Khan University Hospital, Karachi, Pakistan

**Keywords:** Electrocardiography, Arrhythmia, Age, Gender, South Asian race

## Abstract

**Background:**

Little is known about the prevalence of arrhythmias in Pakistan. We assessed the frequency of arrhythmias and electrocardiographic (ECG) abnormalities among the Pakistani population, attending the outpatient department, categorized by age, gender, and geographic region.

**Objective:**

This study aimed to evaluate variations in arrhythmia prevalence and ECG abnormalities across a large sample of a South Asian population.

**Methods:**

This retrospective study analyzed 8746 ECGs from Aga Khan University Hospital in 2022. The ECGs were digitally saved, initially interpreted by an ECG-trained cardiac technician, reviewed by a junior cardiologist using the Minnesota Code, and validated by a senior cardiologist. An electrophysiologist resolved discrepancies to ensure accurate final results.

**Results:**

A total of 8746 ECGs were analyzed (56.86% from men). The prevalences of common arrhythmia were sinus bradycardia 8.53%, sinus tachycardia 5.08%, atrial fibrillation (AF) (1.17%), atrial flutter (AFL) (0.18%), first-degree atrioventricular (AV) block (1.31%), and other AV blocks (0.14%). Women had more sinus tachycardia (6.44%, *P* <.05), AF (1.59%, *P* <.001), and poor R-wave progression (6.41%, *P* <.005), whereas men had more sinus bradycardia (10.68%, *P* <.05), left-anteiror fasicular block (8.32%, *P* <.005), left ventricular hypertrophy (LVH) (2.59%, *P* <.005), pathologic Q waves (3.86%, *P* <.005), and early repolarization pattern (5.87%, *P* <.05). Patients from Khyber Pakhtunkhwa region had higher rates of LVH (7.69%, *P* <.005) and AF (3.85%, *P* <.05). In contrast, patients from Gilgit-Baltistan had the highest rates of sinus bradycardia (28.57%, *P* <.005). Right bundle branch block (10.61%, *P* <.005) and long QT (3.79%, *P* <.005) were more prevalent in the youngest population, whereas patients aged >65 years had more AF (3.31%, *P* <.005), AFL (0.59%, *P* <.005), sinus bradycardia (10.71%, *P* <.001), first-degree AV block (4.62%, *P* <.005), left anterior fascicular block (13.08%, *P* <.005), left bundle branch block (2.43%, *P* <.005), poor R-wave progression (8.82%, *P* <.005), and pathologic Q waves (5.98%, *P* <.005).

**Conclusion:**

This study details important information about arrhythmia prevalence in a large sample of a South Asian population. AF is more common in women, the elderly, and people from Khyber Pakhtunkhwa.

## Graphical abstract

**Figure:**
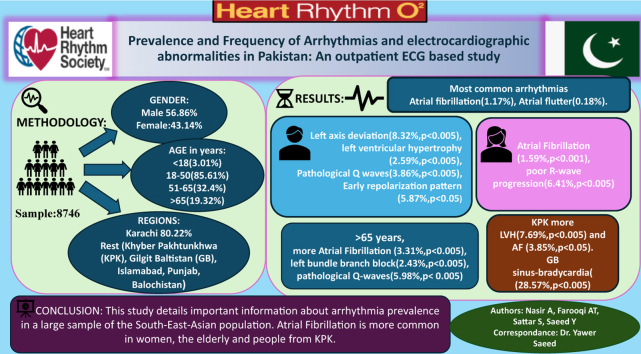

**Key Findings**

▪ The younger population exhibits benign electrocardiographic patterns such as sinus arrhythmias and early repolarization, whereas older adults are prone to more complex conditions such as atrial fibrillation, atrial flutter, and bradyarrhythmia.
▪ Atrial fibrillation is more prevalent in women, with rates similar to that in other Asian populations but lower than in Western populations.
▪ Atrial fibrillation is notably higher in the Khyber Pakhtunkhwa region, whereas bradyarrhythmia is more common in Gilgit-Baltistan.
▪ This first-of-its-kind study provides critical insights into age-, gender-, and region-specific variations, guiding personalized approaches to cardiovascular care in South Asia.

## Introduction

Cardiovascular diseases rank as the primary global cause of mortality and exert a significant socioeconomic influence.^1^, ^2^, ^3^ Given their substantial mortality and morbidity rates, it is imperative to focus on diagnosing and effectively managing cardiovascular diseases in clinical settings. Pakistan is a third-world country with 6 provinces, more than 5 major ethnic groups, and a population of 241.5 million according to a census conducted in 2023.^4^ The World Health Organization’s country profiles for 2019 show that non-communicable diseases account for 58% of all deaths in Pakistan, with ischemic heart disease being the leading cause (15.86%).^5^^,^^6^ Thus, it is essential to screen and manage cardiac diseases in asymptomatic individuals to reduce the future cost of treatment and reduce disease-affected years.

The electrocardiogram (ECG) serves as an economical and widely accessible method for identifying different cardiac abnormalities, with a particular emphasis on rhythm and conduction disturbances. Every parameter of the ECG has been carefully studied. Variables such as PR interval, QRS complex duration, QT interval, distance between each complex, and wave amplitude have been meticulously calculated, and normal reference ranges have been derived.^7^^,^^8^ Any findings beyond this range are considered abnormal and become a characteristic variable indicating a pathology. Each pathology has been shown to have a specific set of characteristics on ECG that differentiate one diagnosis from another.^8^ The prevalences of various arrhythmias and ECG abnormalities have been documented in diverse populations,^9^, ^10^, ^11^ however there is a lack of published data specifically from South Asian populations.

The purpose of this study was to retrospectively evaluate ECG parameters related to age, gender, and different provinces of Pakistan. This would allow us to determine whether there are any age-, gender-, or region-specific variations in the occurrence of arrhythmia and other ECG abnormalities. These reference profiles could be further used to create new guidelines for screening and diagnosing cardiovascular disease through ECG. This is the first study of its kind to be conducted in Pakistan. Our objective was to identify variations in arrhythmia prevalence and ECG parameters across a large sample of a South Asian, more specifically a Pakistani, population.

## Materials and methods

### Study design

We conducted a retrospective observational study utilizing convenience sampling at the Cardiology Section of the Department of Medicine, Aga Khan University Hospital (Karachi, Pakistan). The research reported in this paper adhered to standard guidelines (Helsinki Declaration) on human subjects/data. Because it was a retrospective study, patient consent was waived.

### Operational definitions

The ECG is a test in which leads are placed on various areas of the human body to measure the electrical pulses of the heart.^7^^,^^8^ The 12-lead ECG measures electrical pulses through 12 different leads to enable localization of pathology to a specific heart wall. It consists of 3 bipolar and 3 unipolar limb leads placed on both arms and left leg, and 6 unipolar chest leads positioned on various positions between the fourth and fifth intercostal spaces.^8^^,^^12^ Ethnicity is a group of people who share common social traits and may have similar physical (anthropometric) features.

Trained technicians recorded a standard 12-lead resting ECG in all cases using a digital recorder Norav Medical 1200S Classic PC-ECG machine (Germany) with simultaneous acquisition of 12 leads. The machine was calibrated at 10 mm/mV and recorded at a paper speed of 25 mm/s. Frequency response ranged from 0.05–35 Hz and sampling frequency was 50 Hz, which met the recommended standards for digital ECG acquisition.^12^ The recorded ECGs were digitally saved to a computer. The validity of ECG interpretation was maintained with each ECG initially reviewed by a trained cardiac technician, followed by 2 cardiologists who applied the Minnesota Code and interpreted the ECGs based on the criteria and methods outlined in the Minnesota coding system.^13^ Disagreements, if any, were resolved through consensus with a clinical cardiac electrophysiologist. For example, when applying the Minnesota coding system, we included the voltage criteria for left ventricular hypertrophy from group 2 along with the long QT interval from group 4.

### Inclusion criteria

All patients who underwent ECG assessment at the Aga Khan University Hospital Karachi from January 1, 2022, to December 31, 2022, in the outpatient cardiopulmonary department were included in the study. Aga Khan University Hospital is a quaternary care hospital in Pakistan and receives referrals from throughout the country.

### Exclusion criteria

Patients with incomplete clinical data and no electronic reports of ECG were not included in the study.

### Data collection procedure

Data were collected on a predesigned data entry form after reviewing the electronic medical records of all included patients.

### Data analysis procedure

Data were analyzed using the statistical software package SPSS Version 23 (SPSS Inc., Chicago, IL). In the descriptive analysis, percentages/frequencies were calculated for categorical variables. The χ^2^ test was applied to categorical variables, and analysis of variance was applied to continuous variables (95% confidence interval). *P* <.05 was considered significant.

### Ethical consideration

The study was conducted following approval from the Ethical Review Committee (ERC) of the hospital (ERC Approval Number 5098-11368). The study was conducted in compliance with the protocol and ERC regulatory requirements. Complete privacy was ensured. Patients’ medical record numbers were the only identifiable information that was collected. Only study personnel approved by the institutional review board had access to the data.

## Results

Of the 8746 ECGs analyzed, 56.86% were males, 85.61% were from patients within the age range 18–50 years, and 80.22% originated from the Karachi district. The most common arrhythmias in our population were atrial fibrillation (AF) (1.17%); atrial flutter (AFL) (0.18%); supraventricular tachycardia (SVT) (0.08%); sinus tachycardia (5.08%); sinus arrhythmia (0.79%); sinus bradycardia (8.53%); first-degree atrioventricular (AV) block (1.31%); Mobitz type I, II, and complete heart block (0.14%); and sinus node disease (0.03%). We classified our dataset based on gender, age, and regional differences. Table 1 shows the frequency of various arrhythmias and ECG abnormalities with age that the interpreting cardiologist deemed abnormal (n = 4123 [47.14%]). We divided our population into 4 age groups: <18 years (3.01%); 18–50 years (85.61%); 51–65 years (32.4%); and >65 years (19.32%). Sinus arrhythmia exhibited a higher prevalence in individuals aged <18 years (3.79%, *P* <.005). In addition, this age group showed increased occurrences of sinus tachycardia (8.33%, *P* <.005), right-axis deviation (10.98%, *P* <.005), right bundle branch block (RBBB) (10.61%, *P* <.005), left ventricular hypertrophy (LVH) (4.17%, *P* <.001), right ventricular hypertrophy (5.30%, *P* <.005), and long QT interval (3.79%, *P* <.005). However, patients aged >65 years displayed significantly higher prevalences of AF (3.31%, *P* <.005), AFL (0.59%, *P* <.005), SVT (0.36%, *P* <.005), sinus bradycardia (10.71%, *P* <.001), first-degree AV block (4.62%, *P* <.005), left anterior fascicular block (LAFB) (13.08%, *P* <.005), left bundle branch block (LBBB) (2.43%, *P* <.005), ventricular premature contractions (2.90%, *P* <.005), poor R-wave progression (8.82%, *P* <.005), and pathologic Q waves (5.98%, *P* <.005). Also, the early repolarization pattern was most prevalent among individuals aged 18–50 years (5.46%, *P* = .00).

**Table 1:** ECG parameters with age

	Total (N = 8746)	Age, y				*P* value
		<18 (n = 264)	18–50 (n = 3958)	51–65 (n = 2834)	>65 (n = 1690)	
Normal ECG	4623 (52.86)	174 (65.91)	2390 (60.38)	1455 (51.34)	604 (35.74)	.000
Rhythm						
Sinus with normal HR	8619 (98.55)	261 (98.86)	3937 (99.47)	2801 (98.84)	1620 (95.86)	.000
Sinus arrhythmia	69 (0.79)	10 (3.79)	39 (0.99)	7 (0.25)	13 (0.77)	.000
Ectopic atrial	4 (0.05)	0	1 (0.03)	2 (0.07)	1 (0.06)	.818
Junctional	1 (0.01)	0	0	1 (0.04)	0	.555
Isorhythmic AV dissociation	1 (0.01)	0	1 (0.03)	0	0	.751
Paced	11 (0.13)	0	1 (0.03)	1 (0.04)	9 (0.53)	.000
Tachycardia						
Sinus	444 (5.08)	22 (8.33)	225 (5.68)	128 (4.52)	69 (4.08)	.003
Atrial fibrillation	102 (1.17)	3 (1.14)	16 (0.40)	27 (0.95)	56 (3.31)	.000
Atrial flutter	16 (0.18)	0	2 (0.05)	4 (0.14)	10 (0.59)	.000
SVT	7 (0.08)	0	0	1 (0.04)	6 (0.36)	.000
Bradycardia/AV blocks						
Sinus	746 (8.53)	14 (5.30)	332 (8.39)	219 (7.73)	181 (10.71)	.001
Sinus node disease	3 (0.03)	0	1 (0.03)	2 (0.07)	0	.604
First-degree	115 (1.31)	1 (0.38)	14 (0.35)	22 (0.78)	78 (4.62)	.000
Wenckebach	3 (0.03)	0	0	0	3 (0.18)	.006
Second degree	6 (0.07)	0	0	2 (0.07)	4 (0.24)	.020
High-degree	1 (0.01)	0	0	0	1 (0.06)	.243
Complete heart block	3 (0.03)	0	0	3 (0.11)	0	.100
Axis						
Right-axis deviation	67 (0.77)	29 (10.98)	25 (0.63)	7 (0.25)	6 (0.36)	.000
Left-axis deviation	367 (4.20)	8 (3.03)	82 (2.07)	174 (6.14)	103 (6.09)	.000
Conduction delay						
LAFB	633 (7.24)	1 (0.38)	134 (3.39)	277 (9.77)	221 (13.08)	.000
RBBB	296 (3.38)	28 (10.61)	48 (1.21)	89 (3.14)	131 (7.75)	.000
Incomplete RBBB	184 (2.10)	5 (1.89)	78 (1.97)	67 (2.36)	24 (2.01)	.706
LBBB	67 (0.77)	0	7 (0.18)	19 (0.67)	41 (2.43)	.000
IVCD	36 (0.41)	1 (0.38)	11 (0.28)	12 (0.42)	12 (0.71)	.144
Hypertrophy						
LVH	175 (2.0)	11 (4.17)	57 (1.44)	62 (2.19)	45 (2.66)	.001
RVH	20 (0.23)	14 (5.30)	6 (0.15)	0	0	.000
Premature beats						
APCs	59 (0.64)	0	11 (0.28)	18 (0.64)	27 (1.60)	.000
VPCs	123 (1.41)	0	39 (0.99)	35 (1.24)	49 (2.90)	.000
Miscellaneous						
Poor R wave progression	451 (5.16)	0	136 (3.44)	166 (5.86)	149 (8.82)	.000
ST-segment elevation	11 (0.13)	0	4 (0.10)	6 (0.21)	1 (0.06)	.425
Q waves	251 (2.87)	1 (0.38)	47 (1.19)	102 (3.60)	101 (5.98)	.000
Early repolarization pattern	341 (3.90)	4 (1.52)	216 (5.46)	78 (2.75)	43 (2.54)	.000
Nonspecific ST-T changes	1039 (11.88)	4 (1.52)	413 (10.43)	382 (13.48)	240 (14.20)	.000
Long QT interval	14 (0.16)	10 (3.79)	2 (0.05)	2 (0.07)	0	.000
WPW/pre-excitation	9 (0.10)	0	6 (0.15)	1 (0.04)	2 (0.12)	.478
Low voltage	34 (0.39)	0	10 (0.25)	15 (0.53)	9 (0.53)	.153

Values are given as n (%) unless otherwise indicated.

APC = atrial premature complex; AV = atrioventricular; ECG = electrocardiogram; HR = heart rate; IVCD = interventricular conduction delay; LAFB = left anterior fascicular block; LBBB = left bundle branch block; LVH = left ventricular hypertrophy; RBBB = right bundle branch block; RVH = right ventricular hypertrophy; SVT = supraventricular tachycardia; VPC = ventricular premature complex; WPW = Wolff-Parkinson-White syndrome.

**Figure 1:**
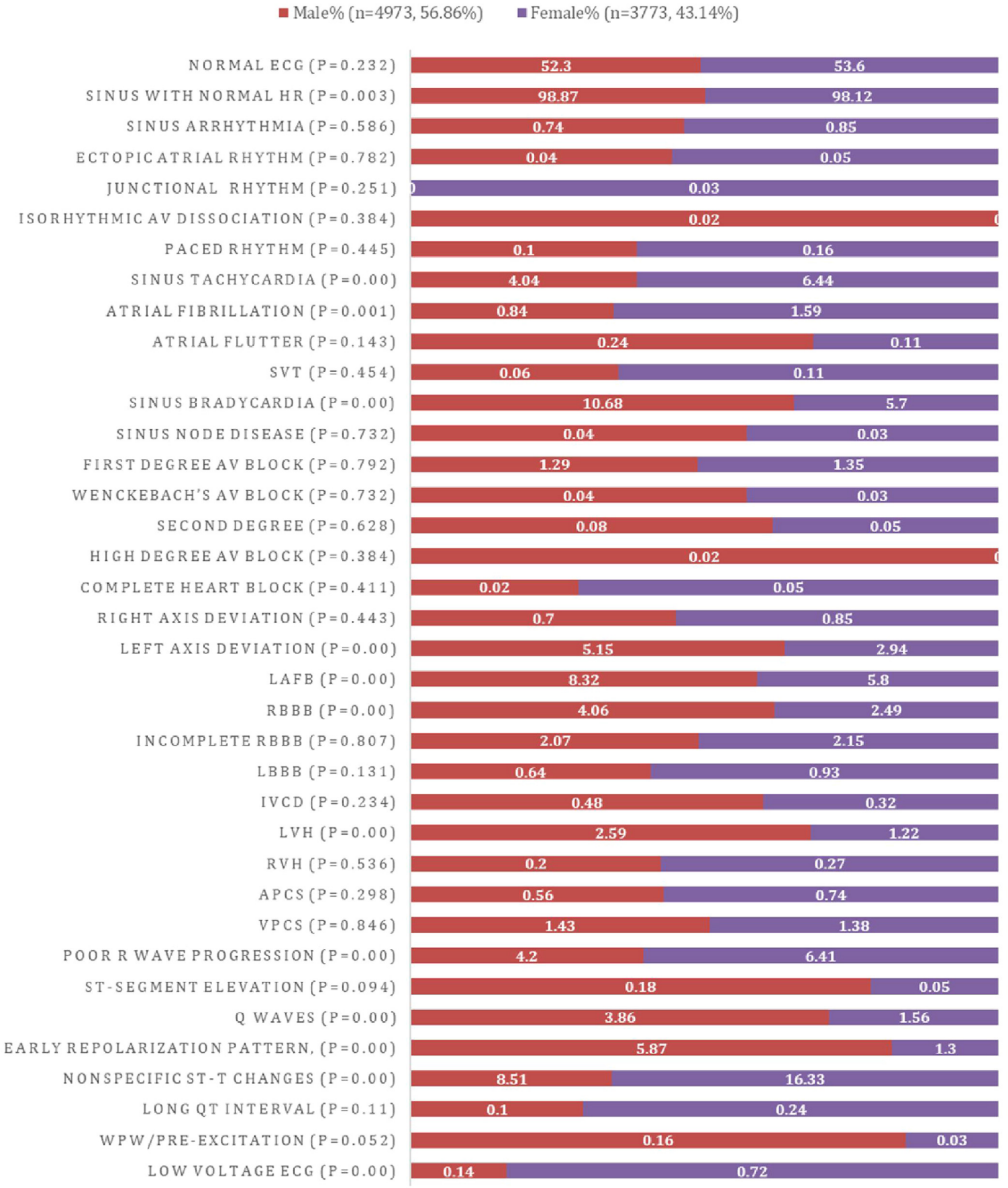
Electrocardiographic (ECG) parameters according to gender. APCS = atrial premature complexes; AV = atrioventricular; HR = heart rate; IVCD = interventricular conduction delay; LAFB = left anterior fascicular block; LBBB = left bundle branch block; LVH = left ventricular hypertrophy; RBBB = right bundle branch block; RVH = right ventricular hypertrophy; SVT = supraventricular tachycardia; VPCS = ventricular premature complexes; WPW = Wolff-Parkinson-White syndrome.

The ECG patterns analyzed concerning gender revealed a prevalence of male dominance in our sample population (56.86%) (Figure 1). Notably, the male population showed higher frequencies of sinus bradycardia (10.68%, *P* = .00), left anterior fascicular block (8.32%, *P* = .00), RBBB (4.06%, *P* = .00), LVH (2.59%, *P* = .00), pathologic Q waves (3.86%, *P* = .00), Wolff-Parkinson-White syndrome/pre-excitation (0.16%, *P* = .052), and early repolarization (5.87%, *P* = .00). In contrast, the female cohort exhibited significantly higher incidences of sinus tachycardia (6.44%, *P* = .00), AF (1.59%, *P* = .001), poor R-wave progression (6.41%, *P* = .00), nonspecific ST-T changes (16.33%, *P* = .00), and low-voltage ECGs (0.72%, *P* = .00).

**Figure 2:**
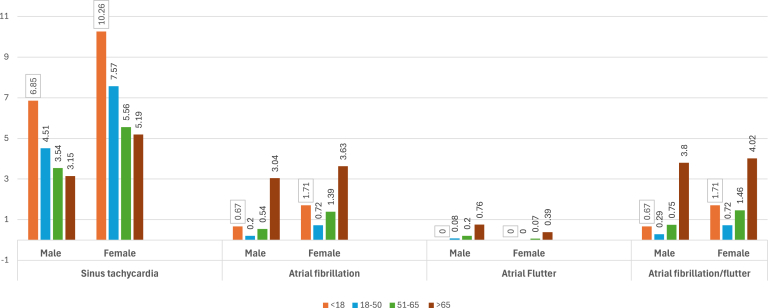
Prevalence (%) of tachycardias according to age and gender.

We also analyzed the prevalence trends of arrhythmias and ECG abnormalities according to the age groups of each gender. Figure 2 illustrates the prevalence of various tachycardias in our population. Sinus tachycardia is most common in the younger age group (18–50 years) for both genders, with its prevalence decreasing as age increases. However, AF and AFL are most prevalent in the elderly population, regardless of gender, with an exponential increase as age advances.

**Figure 3:**
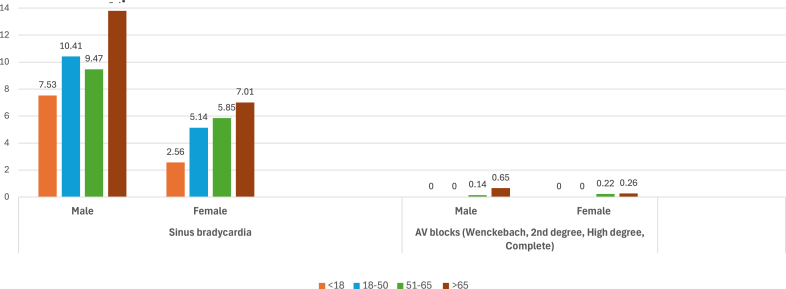
Prevalence (%) of bradycardias according to age and gender. AV = atrioventricular.

Figure 3 shows the trend of bradycardias, with sinus bradycardia and AV blocks most common in elderly >65 years.

**Figure 4:**
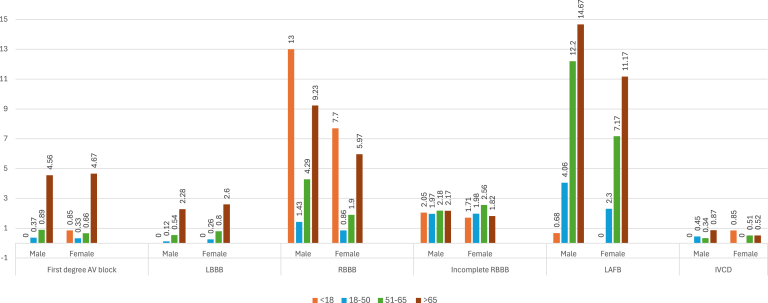
Prevalence (%) of conduction abnormalities according to age and gender. Abbreviations as in Figure 1.

**Figure 5:**
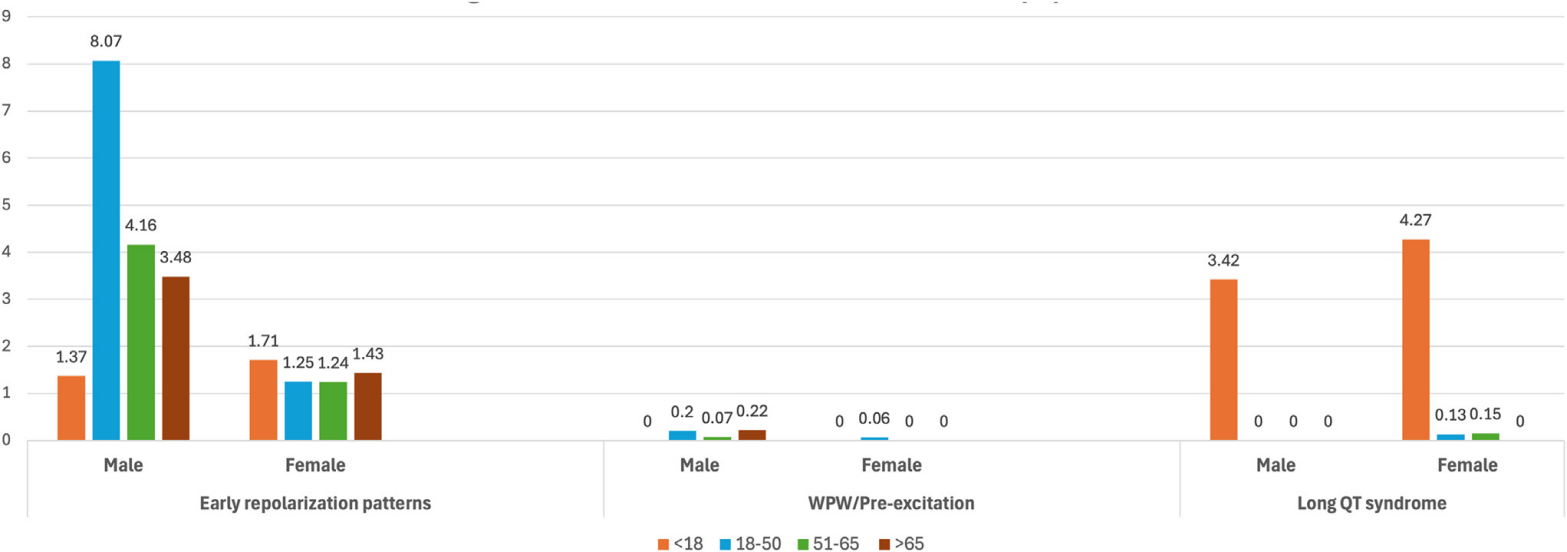
Prevalence (%) of inherited conditions. WPW = Wolff-Parkinson-White syndrome.

Examining various conduction abnormalities, Figure 4 shows that the prevalence of first-degree AV block, LBBB, and LAFB increase with age in both genders. Incomplete RBBB and intraventricular conduction delay are most common in people aged 18–65 years. Although we observed fewer inherited cardiac conditions, Figure 5 shows that early repolarization is most common in individuals aged 18–50 years and decreases with age in both genders. Wolff-Parkinson-White syndrome/pre-excitation also is highest in the age group 18–50 years for both genders, whereas long QT interval is most prevalent in those <18 years, regardless of gender.

**Figure 6:**
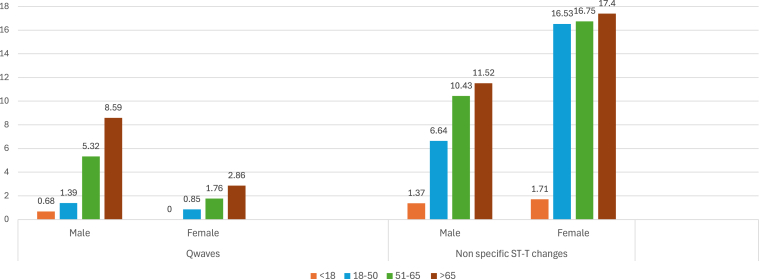
Prevalence (%) of ischemic changes.

Regarding the assessment of ischemic ECG changes, we observed that Q waves and nonspecific ST-T changes are more prevalent in the elderly population >65 years (Figure 6).

**Figure 7:**
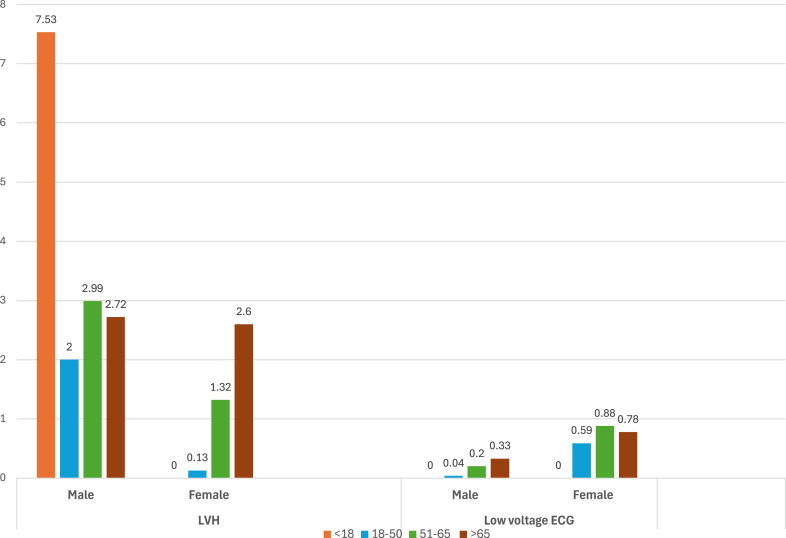
Prevalence (%) of cardiomyopathies. ECG = electrocardiogram; LVH = left ventricular hypertrophy.

Finally, in comparing cardiomyopathies, we noted that LVH is most prevalent in males aged <18 years, decreasing with age (Figure 7). Conversely, there is a linear increase in females, with the highest prevalence found in those >65 years. Low-voltage ECGs show a similar prevalence in the females in age group 51–65 years.

Our data also showed regional differences in arrhythmia prevalence and other abnormalities. As our hospital categorized patients based on their primary residence, most of the participants in our study were from the Karachi district (80.22% [n = 7016]). Consequently, there were no statistically significant regional differences in most ECG parameters. However, notable distinctions were observed. The Khyber Pakhtunkhwa population exhibited the highest prevalence of AF (3.85%, *P* = .01), LVH (7.69%, *P* = .00), and low-voltage ECGs (3.85%, *P* = .01). Conversely, sinus bradycardia (28.57%, *P* = .00) was most observed in patients from Gilgit-Baltistan (Supplemental Materials 1 and 2)

## Discussion

We assessed the occurrences of arrhythmia and other ECG abnormalities in the overall population visiting our outpatient cardiology department through standardized analysis of a substantial collection of ECGs. Our distinctive and extensive data source reveals both similarities and differences compared to previous studies examining ECG abnormalities.^14^, ^15^, ^16^ In our study, we identified AF and AFL in 1.13% of the population, a value consistent with comparable studies involving Asian populations,^15^^,^^17^^,^^18^ in which the prevalence ranged from 0.6%–1.6%. In contrast, in Western populations, the prevalence was higher, ranging from 1.4%–4%.^19^^,^^20^ Our study revealed a lower prevalence of AF compared to other Asian countries and notably less than in Western populations. Of note, the occurrence of AF/AFL increased with age in our cohort and was more prevalent in the female population. Previous studies have shown that AF becomes more common in older adults and is more prevalent in women because of their longer life expectancy. However, the total number of men and women with AF is approximately the same across this elderly population.^21^ Likewise, taking into account a range of risk factors associated with AF,^21^^,^^22^ such as advancing age, obesity, hypertension, diabetes mellitus, hyperthyroidism, alcohol consumption, valvular and structural heart disease, and coronary artery disease, one can infer that the elevated prevalence among older individuals likely is linked to degenerative alterations in cardiac chambers and influenced by the escalating incidence of cardiovascular diseases in Pakistan.^5^^,^^6^

There are some heterogeneous findings regarding gender differences in AF prevalence, but it has been noted that women with AF are more likely to have valvular heart disease, whereas men are at higher risk for coronary artery disease and postoperative AF.^23^ Io addition, rheumatic AF is a significant factor in Asian low-income countries, disproportionately affecting women,^24^ and considering the histologic basis, the onset and persistence of AF are due to significant changes in atrial anatomy and volume.^25^ Similarly, body mass index is a stronger risk factor for AF in men, but other factors such as hypertension and diabetes affect both genders equally. However, Asian women usually are diagnosed with AF at an older age and have higher CHA_2_DS_2_-VASc scores, which often result in a delayed diagnosis of hypertension and the development of diastolic dysfunction.^26^^,^^27^ Pregnancy also increases the risk of AF in women, with the risk increasing with multiparity, likely due to the repeated physiological, inflammatory, and hormonal stresses on the heart, particularly the left atrium.^28^ Nevertheless, our data detected AF in only a single ECG strip, and the history of AF with or without structural heart disease was not considered.

The variations related to age that we identified in our study were consistent with findings from research conducted on both Eastern and Western populations.^14^^,^^15^^,^^17^^,^^19^ These included higher rates of rhythm abnormalities (sinus arrhythmia, sinus tachycardia), conduction abnormalities (right-axis deviation, LBBB), cardiomyopathy (LVH, right ventricular hypertrophy), and inherited condition (long QT interval) in people age <18 years; inherited early repolarization in people between aged 18–50 years; and a substantially higher rate of rhythm abnormalities (AF/AFL, SVT, sinus bradycardia), conduction abnormalities (first-degree AV block, left-axis deviation, LBBB), and ischemic changes (poor R-wave progression and pathologic Q waves) in the elderly population (age >65 years). In our study, rhythm abnormalities were more frequent than conduction abnormalities. Notably, the trend in bradyarrhythmias shows that sinus bradycardia is most commonly observed in young males and young to middle-aged females. However, bradycardia due to AV blocks (second-degree, high-degree, and third-degree) is most common in elderly males but tends to appear earlier in middle-aged females. Hingorani et al^14^ reported ECG abnormalities in 25.5% of the Indian population, with rhythm abnormalities surpassing conduction abnormalities, a pattern consistent with our study. However, their study reported a higher occurrence of the first-degree AV block (2.2% vs 1.31%), whereas our study showed a more frequent presence of complete or incomplete RBBB, LBBB, and LAFB, possibly attributable to a higher age distribution in our study population. Compared to a study by Haatajae et al,^15^ our research exhibited higher prevalences of complete RBBB (1.1% vs 3.38%), incomplete RBBB (1% vs 2.10%), and LAFB (0.1% vs 7.24%) but lower prevalences of LBBB (0.9% vs 0.77%) and intraventricular conduction delay (0.6% vs 0.41%).

Discrepancies in the intracardiac conduction system between genders arise from variations in gene expression originating from sex chromosomes as well as through the impact of sex-related hormones and other environmental factors.^29^ In line with a Danish study,^30^ our research revealed that RBBB was prevalent across all age groups and particularly among males, which has been linked to heightened cardiovascular event risk. Also, our results showed a higher frequency of RBBB in men compared to women. It is widely established that men exhibit higher rates of conduction delays and RBBB compared to women.^31^ This pattern was also observed in our current study concerning conduction abnormalities (LAFB, RBBB), cardiomyopathy(LVH), and ischemic changes (pathologic Q waves). However, this gender disparity was not evident for LBBB, which is more common in females likely because of the higher prevalence of hypertension-related complications.^26^^,^^27^ In our study, the prevalence of LBBB was 0.64% in men and 0.93% in women, although this difference did not reach statistical significance. Other studies have reported similar prevalences of LBBB in both sexes.^32^^,^^33^

When considering inherited conditions, our observations align with previous research showing that these conditions are more prevalent in younger individuals, particularly males.^14^^,^^15^^,^^31^ However, there is a slight exception with the long QT pattern, which tends to occur more often in younger females, although this difference is not statistically significant.

We also evaluated ECG abnormalities associated with ischemia and cardiomyopathies, noting that LVH by voltage criteria is more frequently seen in younger male less than 18 years, with the highest prevalence occurring in elderly females >65 years. The higher incidence of LVH in younger males is supported by various studies and likely is related to the athletic nature of healthy males, whereas in elderly females, LVH is more commonly associated with hypertension.^26^^,^^33^^,^^34^ Additionally, the higher occurrence of abnormalities in females, such as poor R-wave progression, nonspecific ST-T changes, and low-voltage ECGs, aligns with previous research.^20^^,^^35^ Conversely, pathologic Q waves are more common in the middle-aged to elderly populations, particularly among males, consistent with earlier studies.^9^^,^^33^^,^^36^ Moreover, the ischemic changes detected in ECGs among asymptomatic elderly individuals suggest that this noninvasive, affordable, and simple method can be a highly reliable indicator of ischemic heart disease. This is especially true during the sixth and seventh decades of life when the reliability of other factors, such as medical history or symptoms, may decrease.^36^

A Russian study found a correlation between regional variations and the probability of ECG abnormalities, relating to the industrial and economic aspects of living conditions.^37^ Similarly, we observed a few statistically significant regional disparities in ECG abnormalities among the Pakistani populace. Notably, patients form Khyber Pakhtunkhwa exhibits the highest incidence of AF, LVH, and low-voltage ECGs, whereas patients from Gilgit-Baltistan exhibited predominately sinus bradycardia. The elevated prevalence of AF among patients in Khyber Pakhtunkhwa may be linked to increased susceptibility to cardiovascular complications associated with diabetes mellitus, hypertension, and a shared ancestry with European groups.^21^^,^^38^ Conversely, the bradycardia observed in the population of Gilgit-Baltistan can be attributed to their residence at higher altitudes and a notably more active and athletic lifestyle.^39^

### Study strengths and limitations

This study stands as the sole extensive community-based investigation into the prevalence of arrhythmias and ECG abnormalities within a South Asian population. The study encompassed a broad age spectrum and maintained representativeness for the regional population. ECGs were meticulously recorded, stored, and electronically interpreted, with thorough measurements cross-verified by experienced cardiologists. Because Karachi is the largest city in the country and there were no significant differences in ECG abnormalities/arrhythmias across different regions compared to the Karachi district (excluding AF and bradyarrhythmia), our research can serve as a representative of the entire Pakistani population.

Despite these strengths, the study has limitations, such as a modest sample size and a wide but unevenly distributed age range with relatively smaller samples from those <18 years and the elderly population. Additionally, there was a gender and regional distribution imbalance, leaning toward males and the Karachi district. These limitations should be considered when interpreting the findings presented in this study.

## Conclusion

We have conducted a first-of-its-kind study examining variations in ECG parameters within a substantial sample of a South Asian population. The younger demographic is inclined toward sinus arrhythmias, early repolarization, and pre-excitation patterns, whereas the elderly population is more susceptible to AF, AFL, LBBB, and bradyarrhythmia. We observed that AF is more common in women, with its occurrence similar to that seen in the Asian population but lower than the prevalence in Western populations. Moreover, patients from Khyber Pakhtunkhwa exhibited a higher incidence of AF compared to other regions, whereas those from Gilgit-Baltistan were more prone to experiencing bradyarrhythmia.

## References

[1] Vasan R.S., Enserro D.M., Xanthakis V., Beiser A.S., Seshadri S. (2022 26). Temporal trends in the remaining lifetime risk of cardiovascular disease among middle-aged adults across 6 decades: the Framingham study. Circulation.

[2] GBD 2013 Mortality and Causes of Death Collaborators (2015). Global, regional, and national age-sex specific all-cause and cause-specific mortality for 240 causes of death, 1990–2013: a systematic analysis for the Global Burden of Disease Study 2013. Lancet.

[3] Goryakin Y., Suhrcke M. (2014). The prevalence and determinants of catastrophic health expenditures attributable to non-communicable diseases in low- and middle-income countries: a methodological commentary. Int J Equity Health.

[4] Announcement of Results of 7th Population and Housing Census-2023 ‘The Digital Census’. https://www.pbs.gov.pk/sites/default/files/population/2023/Press%20Release.pdf.

[5] (2019). Country profile: Pakistan WHO Data. https://data.who.int/countries/586.

[6] Kazmi T., Nagi M., Razzaq S., Hussnain S., Shahid N., Athar U. (2022). Burden of noncommunicable diseases in Pakistan. East Mediterr Health J.

[7] De Luna A.B. (2019). Willem Einthoven and the ECG. Eur Heart J.

[8] Lux R.L. (2017). Basis and ECG measurement of global ventricular repolarization. J Electrocardiol.

[9] Denes P., Garside D.B., Lloyd-Jones D. (2013). Major and minor electrocardiographic abnormalities and their association with underlying cardiovascular disease and risk factors in Hispanics/Latinos (from the Hispanic Community Health Study/Study of Latinos). Am J Cardiol.

[10] Khurshid S., Choi S.H., Weng L.C. (2018). Frequency of cardiac rhythm abnormalities in a half million adults. Circ Arrhythm Electrophysiol.

[11] Li J., Wang H., Cao C., Xiao C. (2011). Analysis of the 12-leads electrocardiogram of 8970 cases from community natural population. Heart.

[12] Kligfield P., Gettes L.S., Bailey J.J. (2007). Recommendations for the standardization and interpretation of the electrocardiogram: part I: The electrocardiogram and its technology: a scientific statement from the American Heart Association Electrocardiography and Arrhythmias Committee, Council on Clinical Cardiology; the American College of Cardiology Foundation; and the Heart Rhythm Society: endorsed by the International Society for Computerized Electrocardiology. Circulation.

[13] Prineas R.J., Crow R.S., Zhang Z. (2010).

[14] Hingorani P., Natekar M., Deshmukh S. (2012). Morphological abnormalities in baseline ECGs in healthy normal volunteers participating in phase I studies. Indian J Med Res.

[15] Haataja P., Nikus K., Kähönen M. (2013). Prevalence of ventricular conduction blocks in the resting electrocardiogram in a general population: the Health 2000 Survey. Int J Cardiol.

[16] Saggu D.K., Sundar G., Nair S.G. (2016). Prevalence of atrial fibrillation in an urban population in India: the Nagpur pilot study. Heart Asia.

[17] Iguchi Y., Kimura K., Aoki J. (2008). Prevalence of atrial fibrillation in community-dwelling Japanese aged 40 years or older in Japan: analysis of 41,436 non-employee residents in Kurashiki-city. Circ J.

[18] Zhou Z., Hu D. (2008). An epidemiological study on the prevalence of atrial fibrillation in the Chinese population of mainland China. J Epidemiol.

[19] Naccarelli G.V., Varker H., Lin J., Schulman K.L. (2009). Increasing prevalence of atrial fibrillation and flutter in the United States. Am J Cardiol.

[20] Marcolino M.S., Palhares D.M., Benjamin E.J., Ribeiro A.L. (2015). Atrial fibrillation: prevalence in a large database of primary care patients in Brazil. Europace.

[21] Schnabel R.B., Yin X., Gona P. (2015). 50 year trends in atrial fibrillation prevalence, incidence, risk factors, and mortality in the Framingham Heart Study: a cohort study. Lancet.

[22] Vermond R.A., Geelhoed B., Verweij N. (2015). Incidence of atrial fibrillation and relationship with cardiovascular events, heart failure, and mortality: a community-based study from The Netherlands. J Am Coll Cardiol.

[23] Wolbrette D., Naccarelli G., Curtis A., Lehmann M., Kadish A. (2002). Gender differences in arrhythmias. Clin Cardiol.

[24] Sharma S.K., Verma S.H. (2015). A clinical evaluation of atrial fibrillation in rheumatic heart disease. J Assoc Physicians India.

[25] Noubiap J.J., Nyaga U.F., Ndoadoumgue A.L., Nkeck J.R., Ngouo A., Bigna J.J. (2020). Meta-analysis of the incidence, prevalence, and correlates of atrial fibrillation in rheumatic heart disease. Glob Heart.

[26] Ullah H., Khan S.B., Khan S.S., Khan Z.A., Shah I., Hafizullah M. (2012). Gender differences in left ventricular diastolic dysfunction in normotensive type 2 diabetic patients. Pak Heart J.

[27] Regitz-Zagrosek V., Brokat S., Tschope C. (2007). Role of gender in heart failure with normal left ventricular ejection fraction. Prog Cardiovasc Dis.

[28] Wong J.A., Rexrode K.M., Sandhu R.K., Conen D., Albert C.M. (2017). Number of pregnancies and atrial fibrillation risk: The women's health study. Circulation.

[29] Garcia M., Mulvagh S.L., Merz C.N., Buring J.E., Manson J.E. (2016). Cardiovascular disease in women: clinical perspectives. Circ Res.

[30] Bussink B.E., Holst A.G., Jespersen L., Deckers J.W., Jensen G.B., Prescott E. (2013). Right bundle branch block: prevalence, risk factors, and outcome in the general population: results from the Copenhagen City Heart Study. Eur Heart J.

[31] Kreger B.E., Anderson K.M., Kannel W.B. (1989). Prevalence of intraventricular block in the general population: the Framingham Study. Am Heart J.

[32] Imanishi R., Seto S., Ichimaru S., Nakashima E., Yano K., Akahoshi M. (2006). Prognostic significance of incident complete left bundle branch block observed over a 40-year period. Am J Cardiol.

[33] De Bacquer D., De Backer G., Kornitzer M. (2000). Prevalences of ECG findings in large population-based samples of men and women. Heart.

[34] Pickham D., Zarafshar S., Sani D., Kumar N., Froelicher V. (2014). Comparison of three ECG criteria for athlete pre-participation screening. J Electrocardiol.

[35] Chugh S.S., Havmoeller R., Narayanan K. (2014). Worldwide epidemiology of atrial fibrillation: a Global Burden of Disease 2010 Study. Circulation.

[36] Erelund S., Karp K., Wiklund U., Hörnsten R., Arvidsson S. (2021;May). Are ECG changes in heart-healthy individuals of various ages related to cardiac disease 20 years later?. Ups J Med Sci.

[37] Maksimov S., Muromtseva G., Kutsenko V., Shalnova S., Evstifeeva S., Drapkina O. (2023). Major and minor ECG abnormalities depending on regional living conditions in Russia. Sci Rep.

[38] Riaz S, Naz S, Kousar U, Malik ZEH, Bashir R. Major complication associated with diabetes mellitus type II in Punjab and Khyber Paktunkhwa population. J Diabetes Metab 20189:809.

[39] Berthelsen L.F., van Diepen S., Steele A.R. (2022). Duration at high altitude influences the onset of arrhythmogenesis during apnea. Eur J Appl Physiol.

