# Supplementary material for: Prevalence and frequency of arrhythmias and electrocardiographic abnormalities in Pakistan: An outpatient ECG-based study
Source: Heart Rhythm O2. 2025 Jan 16;6(4):489–98. doi: 10.1016/j.hroo.2024.12.017 (PMC12047508; doi:10.1016/j.hroo.2024.12.017)
Supplement: Supplemental Table [file mmc1.docx]

**Supplementary material 1:** Regional variations in ECG parameters

|  | Total (n=8746) | Karachi (n=7016) | Sindh(n=740) | Punjab(n=175) | Balochistan(n=526) | KPK (n=52) | ISL(n=54) | GB (n=42) | N.A (n=141) | p-value |  |
| --- | --- | --- | --- | --- | --- | --- | --- | --- | --- | --- | --- |
| Normal ECG | 4623(52.86%) | 3741(53.32%) | 380(51.35%) | 92(52.57%) | 261(49.62%) | 30(57.90%) | 24(44.44%) | 18(42.86%) | 77(54.61%) | 0.391 |  |
| Rhythm | | | | | | | | | | | |
| Sinus with normal HR | 8619(98.55%) | 6924(98.69%) | 729(87.51%) | 173(98.86%) | 510(96.96%) | 50(96.15%) | 53(98.15%) | 42(100%) | 138(98.87%) | 0.059 |  |
| Sinus arrhythmia | 69 (0.79%) | 58(0.83%) | 6(0.81%) | 1(0.57%) | 4(0.376%) | 0 | 0 | 0 | 0 | 0.924 |  |
| Ectopic atrial | 4(0.05%) | 2(0.03%) | 1(0.14%) | 0 | 1(0.19%) | 0 | 0 | 0 | 0 | 0.737 |  |
| Junctional | 1(0.01%) | 1(0.01 | 0 | 0 | 0 | 0 | 0 | 0 | 0 | 1.0 |  |
| Isorhythmic AV dissociation | 1(0.01%) | 1(0.01%) | 0 | 0 | 0 | 0 | 0 | 0 | 0 | 1.0 |  |
| Paced | 11(0.13%) | 11(0.16%) | 0 | 0 | 0 | 0 | 0 | 0 | 0 | 0.910 |  |
| Tachycardia | | | | | | | | | | | |
| Sinus | 444(5.08%) | 342(4.87%) | 53(7.16%) | 6(3.43%) | 32(6.08%) | 2(3.85%) | 4(7.41%) | 0 | 5(3.55%) | 0.070 |  |
| Atrial fibrillation | 102 (1.17%) | 71(1.01%) | 9(1.22%) | 2(1.14%) | 15(2.85%) | 2(3.85%) | 1(1.85%) | 0 | 2(1.42%) | 0.010 |  |
| Atrial Flutter | 16 (0.18%) | 16(0.23%) | 0 | 0 | 0 | 0 | 0 | 0 | 0 | 0.785 |  |
| SVT | 7(0.08%) | 6(0.09%) | 0 | 1(0.57%) | 0 | 0 | 0 | 0 | 0 | 0.477 |  |
| Bradycardia/AV blocks | | | | | | | | | | | |
| Sinus | 746 (8.53%) | 575(8.20%) | 54(7.30%) | 21(12.0%) | 53(10.08%) | 7(13.46%) | 13(24.07%) | 12(28.57%) | 11(7.80%) | 0.000 |  |
| Sinus node disease | 3(0.03%) | 3(0.04%) | 0 | 0 | 0 | 0 | 0 | 0 | 0 | 0.998 |  |
| First degree | 115(1.31%) | 99(1.41%) | 7(0.95%) | 1(0.57%) | 4(0.76%) | 0 | 0 | 1(2.38%) | 3(2.13%) | 0.567 |  |
| Wenckebach’s | 3(0.03%) | 3(0.04%) | 0 | 0 | 0 | 0 | 0 | 0 | 0 | 0.998 |  |
| Second degree | 6(0.07%) | 6(0.09%) | 0 | 0 | 0 | 0 | 0 | 0 | 0 | 0.983 |  |
| High degree | 1(0.01%) | 1(0.01%) | 0 | 0 |  | 0 | 0 | 0 | 0 | 1.0 |  |
| Complete heart block | 3(0.03%) | 3(0.04%) | 0 | 0 | 0 | 0 | 0 | 0 | 0 | 0.998 |  |
| Axis | | | | | | | | | | | |
| Right Axis deviation | 67(0.77%) | 52(0.74%) | 2(0.27%) | 3(1.71%) | 8(1.52%) | 0 | 1(1.85%) | 0 | 1(0.71%) | 0.187 |  |
| Left Axis deviation | 367(4.20%) | 296(4.22%) | 35(4.73%) | 5(2.86%) | 17(3.23%) | 1(1.92%) | 2(3.70%) | 1(2.37%) | 10(7.09%) | 0.481 |  |
| Conduction delay | | | | | | | | | | | |
| LAFB | 633(7.24%) | 506(7.21%) | 62(8.38%) | 13(7.43%) | 30(5.70%) | 1(1.92%) | 6(11.11%) | 2(4.76%) | 13(9.22%) | 0.342 |  |
| RBBB | 296(3.38%) | 255(3.63%) | 20(2.70%) | 2(1.14%) | 12(2.28%) | 1(1.92%) | 2(3.70%) | 1(2.38%) | 3(2.13%) | 0.315 |  |
| Incomplete RBBB | 184(2.10%) | 151(2.15%) | 19(2.57%) | 5(2.86%) | 8(1.52%) | 0 | 1(1.85%) | 0 | 0 | 0.401 |  |
| LBBB | 67(0.77%) | 59(0.84%) | 2(0.27%) | 0 | 4(0.76%) | 0 | 1(1.85%) | 0 | 1(0.71%) | 0.560 |  |
| IVCD | 36(0.41%) | 26(0.37%) | 3(0.41%) | 3(1.71%) | 4(0.76%) | 0 | 0 | 0 | 0 | 0.173 |  |
| Hypertrophy | | | | | | | | | | | |
| LVH | 175(2.0%) | 115(1.64%) | 25(3.38%) | 5(2.86%) | 14(2.66%) | 4(7.69%) | 3(5.56%) | 2(4.76%) | 7(4.96%) | 0.000 |  |
| RVH | 20(0.23%) | 14(0.20%) | 1(0.14%) | 2(1.14%) | 2(0.38%) | 0 | 0 | 0 | 1(0.71%) | 0.235 |  |
| Premature beats | | | | | | | | | | | |
| APCs | 59(0.64%) | 49(0.70%) | 6(0.81%) | 3(1.71%) | 1(0.19%) | 0 | 0 | 0 | 0 | 0.614 |  |
| VPCs | 123(1.41%) | 95(1.35%) | 12(1.62%) | 3(1.71%) | 10(1.90%) | 0 | 0 | 0 | 3(2.13%) | 0.771 |  |
| Miscellaneous | | | | | | | | | | | |
| Poor R wave progression | 451(5.16%) | 375(5.34%) | 37(5.0%) | 6(3.43%) | 27(5.13%) | 1(1.92%) | 1(1.85%) | 1(2.38%) | 3(2.13%) | 0.404 |  |
| ST-segment elevation | 11(0.13%) | 7(0.10%) | 3(0.41%) | 0 | 0 | 0 | 0 | 0 | 1(0.71%) | 0.196 |  |
| Q waves | 251(2.87%) | 190(2.71%) | 28(3.78%) | 7(4.0%) | 20(3.80%) | 2(3.85%) | 1(1.85%) | 1(2.38%) | 2(1.42%) | 0.450 |  |
| Early repolarization pattern | 341(3.90%) | 257(3.66%) | 30(4.05%) | 8(4.57%) | 34(6.46%) | 3(5.77%) | 2(3.70%) | 2(4.76%) | 5(3.55%) | 0.132 |  |
| Nonspecific ST-T changes | 1039(11.88%) | 831(11.84%) | 92(12.43%) | 16(9.14%) | 66(12.55%) | 8(15.38%) | 4(7.41%) | 4(9.52%) | 18(12.77%) | 0.817 |  |
| Long QT syndrome | 14(0.16%) | 12(0.71%) | 1(0.14%) | 1(0.57%) | 0 | 0 | 0 | 0 | 0 | 0.862 |  |
| WPW/Pre-excitation | 9(0.10%) | 7(0.10%) | 1(0.14%) | 0 | 0 | 0 | 1(1.85%) | 0 | 0 | 0.017 |  |
| Low voltage | 34(0.39%) | 32(0.46%) | 0 | 0 | 0 | 2(3.85%) | 0 | 0 | 0 | 0.001 |  |
| ECG = Electrocardiogram; HR = Heart rate; AV= Atrioventricular; SVT = Supraventricular tachycardia; LAFB = Left anterior fascicular block; RBBB = Right bundle branch block; LBBB = Left bundle branch block; IVCD = Intraventricular conduction delay; LVH = Left ventricular hypertrophy; RVH = Right ventricular hypertrophy; APCs = Atrial premature complexes; VPCs = Ventricular premature complexes | | | | | | | | | | |  |
