# Supplementary material for: Prevalence and frequency of arrhythmias and electrocardiographic abnormalities in Pakistan: An outpatient ECG-based study
Source: Heart Rhythm O2. 2025 Jan 16;6(4):489–98. doi: 10.1016/j.hroo.2024.12.017 (PMC12047508; doi:10.1016/j.hroo.2024.12.017)
Supplement: Supplementary Material [file mmc2.pdf]

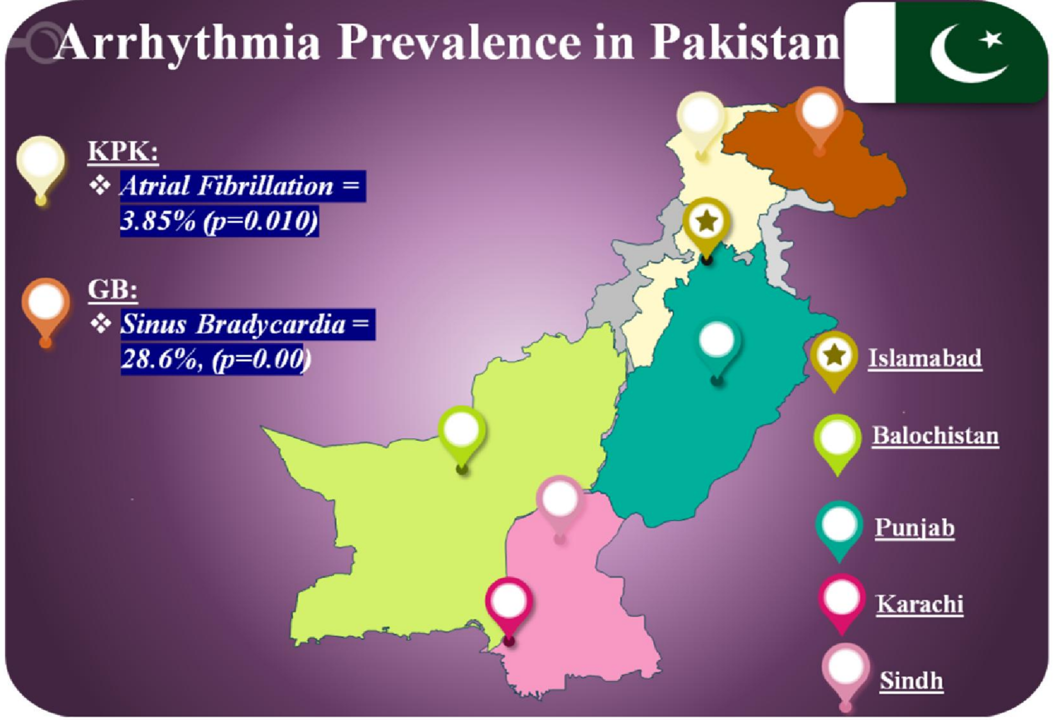

Supplementary material 2: The representation of Pakistan's map, showcasing the prevalence of the most common arrhythmia across various regions
